# Sleep duration, hypnotic drug use, and risk factors: cross- sectional study

**DOI:** 10.1038/s41598-023-30501-6

**Published:** 2023-03-01

**Authors:** Nazanin Jalali, Parvin Khalili, Zahra Jamali, Zahra Jalali, Amir Moghadam-Ahmadi, Alireza Vakilian, Fatemeh Ayoobi

**Affiliations:** 1 Non-Communicable Diseases Research Center, Rafsanjan University of Medical Sciences, Rafsanjan, Iran; 2 Neurology Department, School of Medicine, Rafsanjan University of Medical Sciences, Rafsanjan, Iran; 3 Social Determinants of Health Research Center, Rafsanjan University of Medical Sciences, Rafsanjan, Iran; 4 Department of Epidemiology, School of Public Health, Rafsanjan University of Medical Sciences, Rafsanjan, Iran; 5 Clinical Research Development Unit (CRDU), Niknafs Hospital, Rafsanjan University of Medical Sciences, Rafsanjan, Iran; 6 Department of Clinical Biochemistry, School of Medicine, Rafsanjan University of Medical Sciences, Rafsanjan, Iran; 7 Neuro-Immunology Research Scholar, Neurological Research Laboratory, Jefferson Hospital for Neuroscience, Thomas Jefferson University, Philadelphia, PA USA; 8 Occupational Safety and Health Research Center, NICICO, World Safety Organization and Rafsanjan University of Medical Sciences, Rafsanjan, Iran

**Keywords:** Neuroscience, Physiology, Diseases, Medical research

## Abstract

Both short sleep duration (SSD) and long sleep duration (LSD) are associated with an increased risk of morbidity and mortality. Here, we aimed to assess the prevalence of sleep duration disturbances among adults in association with demographic, medication use, personal habits, and chronic diseases, while also considering the impact of hypnotic drug use. We performed a cross-sectional study of 9991 adult participants of the Rafsanjan Cohort Study (RCS), as part of the Prospective epidemiological research studies in Iran (PERSIAN). Multivariate logistic regression analyses were conducted to assess the association between short (< 6 h) and long (> 9 h) sleep duration with demographic and lifestyle parameters and common non-communicable diseases. Additionally, we performed stratified analysis to investigate the association of sleep duration with the abovementioned factors and diseases, in groups with and without hypnotic drug use. We found higher odds of SSD significantly associated with age (P < 0.001), BMI (P < 0.001), physical activity (P < 0.001), and depression (P = 0.023). LSD displayed a positive association with the female sex (P < 0.001), opium consumption (P < 0.001), and history of MI (P = 0.045), and a reverse connection with education (P = 0.007), physical activity (P < 0.001) and alcohol consumption (P = 0.027). Stratifying for the hypnotic drug use, our sensitivity analyses indicated that in hypnotic drug users, education (P = 0.034) and physical activity (P < 0.001) were associated with LSD, in this group, significantly increased odds ratio of LSD were associated with opium consumption (P = 0.046) and thyroid dysfunction (P = 0.037). Our findings demonstrated the demographic and lifestyle factors and diseases associated with long and short sleep duration in the population of the RCS. Additionally, after stratifying for hypnotic drug use, our results indicated that some diseases are only associated with abnormal sleep duration upon using hypnotic drugs.

## Introduction

Sleep as a systematic biological rhythm-based behavior has a significant role in the revival of daily mental and physiological abilities. Normal sleep contributes significantly to the maintenance of psychological and physical health and recovery from illnesses^1^. The consensus published by the American Academy of Sleep Medicine (AASM) and Sleep Research Society (SRS) in 2015 defined normal sleep duration (NSD) as sleeping more than 6 and less than 9 h per night^2^. During the last half-century, the duration of night-time sleep has decreased by around 1.5–2 h per night, which points to a significant increase in chronic sleep deprivation among adults^3,4^. For example, about 30% of adults in the United States sleep less than 6 h at night and struggle with sleep insufficiency^5^.

Multiple published studies have suggested an association between abnormal sleep duration and increased risk of metabolic syndrome^6,7^, diabetes mellitus^8^, cancer^9^, stroke, cardiovascular diseases, and all-cause mortality^10^. The suggested underlying mechanism explained for this association is the critical role of enough sleep in energy homeostasis in the body, regulating the balance between energy intake and consumption^11^. According to previous studies, obesity is one of the adverse health consequences of sleep deprivation^12^. Another underlying mechanism is attributed to a hormonal disbalance in the body, such as the reported decreased serum level of adiponectin and leptin upon acute or chronic sleep deprivation^13^. Additionally, the fatigue caused by abnormal sleep duration resulted in more energy intake and increased eating times, concurrent with lower physical activity and energy consumption, which consequently leads to obesity, cardiovascular problems, and other related diseases^14^.

Despite statements supporting an adverse effect of abnormal sleep duration on cardiometabolic risk factors and diseases, some contradictory reports, especially on long sleep duration (LSD), did not find a significant association between abnormal sleep duration and cardiovascular and metabolic diseases^15–18^, which warrant further study on this subject.

Pharmacological agents and non-pharmacological agents are prescribed as treatment options for chronic and acute insomnia. While some pharmacological agents, specifically benzodiazepines (BDZ) and non-benzodiazepines (non-BDZ), are recommended by the US National Institute of Health for the management of acute insomnia, their administration for chronic insomnia is under debate due to their side effects, such as physical dependence and withdrawal symptoms, returning insomnia, and long-term safety issues^19^. Hypnotic drug use itself is suggested to be associated with cardiovascular diseases and all-cause mortality by some studies^20–22^. Therefore, it is necessary for studies that investigate the link between abnormal sleep duration with chronic diseases to consider the impact of hypnotic drugs in their analysis. This critical factor is not addressed correctly in most previous studies.

In the current study, we aimed to assess the abnormal sleep duration among adult participants of the Rafsanjan Cohort Study (RCS)^23^. In addition, we sought to determine the relationship between abnormal sleep duration with various non-communicable diseases, such as metabolic and cardiovascular problems, also demographic factors, and some lifestyle parameters such as physical activity and substance use (cigarette, alcohol, and opioid). The use of hypnotic treatments for abnormal sleep has been poorly studied regarding its impact on sleep duration-associated diseases and risk factors. To investigate how pharmaceutical treatment agents may impact sleep duration concerning its predisposing factors and its associated diseases, here we assessed these connections separately in individuals who used hypnotic drugs or not.

## Methods

The current study was performed on 9991 participants of both genders aged 35–70 years old in the RCS^23^ which was a part of the Prospective Epidemiological Research Studies in Iran (PERSIAN)^24^. The RCS conducted in Rafsanjan city in the southeast of Iran was designed to recruit 10,000 participants from Rafsanjan’s urban and suburban areas. Recruitment was performed by a random selection approach via systematic clustering using household numbers. According to the PERSIAN Cohort Central Scientific Committee, the estimated sample size for RCS supports adequate statistical power^23^. Participation in the RCS was voluntary and upon signature of an informed consent form, and the confidentiality of the personal data of the participants was ensured by all necessary measures. Of all the participants, after excluding subjects with incomplete sleep habits questionnaire, 9981 entered our study. The protocol and questionnaires of this cross-sectional study were designed following the Persian cohort study protocols and under the supervision of the Iranian Ministry of Health and Medical Education (IMHME). In addition, they have been approved by the Ethics Committee of Rafsanjan University of Medical Sciences with the Ethical code of IR.RUMS.REC.1398.140, and all methods were carried out followed the relevant guidelines and regulations.

### Data collection

Participants' demographic information was collected by a questionnaire including age, gender, wealth status index (WSI), education levels (The number of years the participant received education), etc. Other data collection included past medical history, anthropometry (height, waist circumference, hip circumference, waist circumference, weight, and BMI), physical activity (Metabolic equivalent of task: MET), medication use (past and present), and personal habits (smoking, opium, and alcohol consumption). All questionnaires prepared in the Farsi language were previously validated in the PERSIAN cohort study^24,25^.

MET is the daily physical activity of the participants, was weighted based on its relative metabolic cost, and MET-h/day for 24 h was derived in this way.

WSI was estimated by multiple correspondence analysis (MCA) of the economic variables. After this step, the subjects were categorized into four groups, including low class (≤ − 0.606), low-middle class (− 0.607–0.0349), middle-high class (0.035–1.169), and high class (≥1.170) based on the 25th, 50th and 90th percentiles.

Based on the Third Report of the National Cholesterol Education Program (NCEP-Adult Treatment Panel III), we defined dyslipidemia as LDL ≥ 130 mg/dL, or TC ≥ 200 mg/dL, or HDL ≤ 40 mg/dL in men, and 50 mg/dl in women or TG ≥ 150 mg/dL and or using lipid-lowering medications during the past 2 weeks^26^.

### Sleep parameters assessment

Sleep habits of the population were assessed using several questions from the Pittsburgh questionnaire. In the present study, total sleep duration was a sum of sleep duration at night and daytime napping sleep hours. Entire sleep duration was classified into three groups: short sleep duration (SSD) (< 6 h), normal sleep duration (NSD) (6–9 h), and long sleep duration (LSD) (> 9 h)^27^. In the multivariable analysis, the 6–9 h category was selected as the reference. The frequency difference between the total number and some covariates was related to missing data.

### Statistical analysis

Quantitative variables were described as mean ± standard deviation, and categorical variables as the frequency and percentage. Also, baseline characteristics and distribution of diseases of individuals were compared across the groups taking or non-taking hypnotic drugs by sleep duration using the chi-square test for categorical variables and the one-way ANOVA test for quantitative variables. The odds ratio (OR: with 95% CIs) of taking hypnotic drugs and non-taking hypnotic drugs based on sleep habits were evaluated by a multinomial logistic regression model and confounder's variables were identified using relevant epidemiological texts and based on subject matter knowledge. Potential confounding variables were sequentially entered into the model according to their hypothesized strengths of association with sleep duration. To reach this goal, confounding variables with a P-value < 0.25 were selected as confounders. All analyses were performed through State V.12. All P-values are two-sided, and P-values < 0.05 and 95% confidence intervals were considered statistically significant.

### Ethical approval

The Ethics Committee of Rafsanjan University of Medical Sciences approved this study (Ethical codes: ID: IR.RUMS.REC. 1398.140). Written informed consent was obtained from the participants. The participant's data were kept confidential and only accessible to the study investigators.

## Result

**Figure 1:**
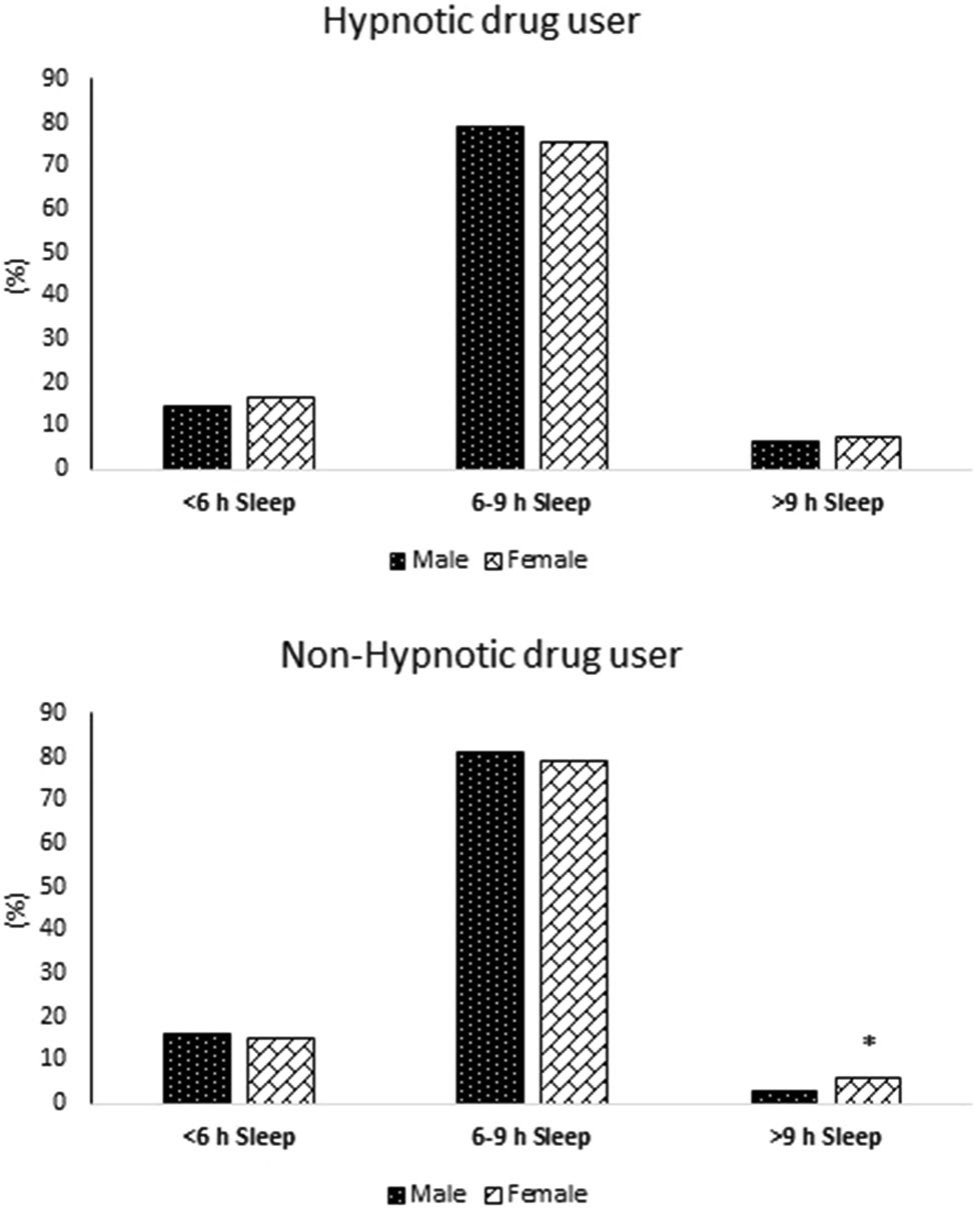
Prevalence of sleep duration in male and female participants based on the use of the hypnotic drugs or not. *P < 0.001.

The demographic characteristics of the participants are presented in Table 1. Among 9981 participants, 1105 (11.07%) were hypnotic drug users (HDU), and 8876 (88.93%) were non-hypnotic drug users (NHDU). LSD (> 9 h) was significantly more common in women in the NHDU group (P < 0.001), but no significant difference was observed in the frequency of SSD or LSD between men and women in the HDU group (Fig. 1). In the total population and NHDU group, LSD (> 9 h) had a significantly higher mean of age and lower mean of WSI (P < 0.001), and also SSD (< 6 h) had a significantly higher mean of BMI (P < 0.001). In the total population, HDU and NHDU groups, LSD (> 9 h) had a significantly lower mean of education, physical activity, and alcohol consumption (P < 0.05), and also NSD (6–9 h) had a significantly lower mean of waist circumference (P < 0.001).

**Table 1:** Baseline characteristics of subjects taking hypnotic drugs or non, by sleep duration in Rafsanjan Cohort Study (RCS).

Variable	Sleep duration											
	Total (n = 9981)				Hypnotic drug user (n = 1105)				Non-hypnotic drug user (8876)			
	< 6 h (n = 1552)	6–9 h (n = 7959)	> 9 h (n = 470)	P-value	< 6 h (n = 177)	6–9 h (n = 848)	> 9 h (n = 80)	P-value	< 6 h (n = 1375)	6–9 h (n = 7111)	> 9 h (n = 390)	P-value
Age- yr. no. (%)												
35–45	483 (31.12)	3107 (39.04)	134 (28.51)	< 0.001	31 (17.51)	179 (21.11)	13 (16.25)	0.660	452 (32.87)	2928 (41.18)	121 (31.03)	< 0.001
46–55	501 (32.28)	2437 (30.62)	131 (27.87)		63 (35.59)	298 (35.14)	32 (40)		438 (31.85)	2139 (30.08)	99 (25.38)	
≥ 56	568 (36.60)	2414 (30.33)	205 (43.62)		83 (46.89)	371 (43.75)	35 (43.75)		485 (35.27)	2043 (28.73)	170 (43.59)	
Mean ± SD	51.13 ± 9.34	49.53 ± 9.54	52.53 ± 10.02	< 0.001	54.29 ± 8.52	53.30 ± 8.98	54.64 ± 9.41	0.22	50.73 ± 9.37	49.08 ± 9.50	52.10 ± 10.09	< 0.001
Gender- no. (%)												
Female	803 (51.74)	4199 (52.76)	328 (69.79)	< 0.001	122 (68.93)	554 (65.33)	56 (70.00)	0.499	681 (49.53)	3645 (51.26)	272 (69.74)	< 0.001
Male	749 (48.26)	3760 (47.24)	142 (30.21)		55 (31.07)	294 (34.67)	24 (30.00)		694 (50.47)	3466 (48.74)	118 (30.26)	
WSI- no. (%)												
Low	367 (23.66)	1832 (23.02)	144 (30.70)	< 0.001	46 (25.99)	209 (24.65)	23 (28.75)	0.854	321 (23.36)	1623 (22.83)	121 (31.11)	< 0.001
Low-middle	410 (26.43)	2293 (28.82)	159 (33.90)		52 (29.38)	268 (31.60)	28 (35.00)		358 (26.06)	2025 (28.49)	131 (33.68)	
Middle-high	654 (42.17)	3196(40.17)	147 (31.34)		69 (38.98)	317 (37.38)	26 (32.50)		585 (42.58)	2879 (40.50)	121 (31.11)	
High	120 (7.74)	636 (7.99)	19 (4.05)		10 (5.65)	54 (6.37)	3 (3.75)		110 (8.01)	582 (8.19)	16 (4.11)	
Mean ± SD	0.008 ± 1.029	0.016 ± 0.99	− 0.290 ± 0.99	< 0.001	-0.171 ± 1.026	− 0.0881.003	− 0.275 ± 1.020	0.21	0.031 ± 1.028	0.028 ± 0.990	− 0.293 ± 0.988	< 0.001
Education. year- no. (%)												
≤ 5	556 (35.82)	2700 (33.93)	238 (50.64)	< 0.001	94 (53.11)	349 (41.16)	51 (63.75)	< 0.001	462 (33.60)	2351 (33.07)	187 (47.95)	< 0.001
6–12	735 (47.36)	3915 (49.20)	195 (41.49)		68 (38.42)	396 (46.70)	24 (30.00)		667 (48.51)	3519 (49.49)	171 (43.85)	
≥ 13	261 (16.82)	1343 (16.88)	37 (7.87)		15 (8.47)	103 (12.15)	5 (6.25)		246 (17.89)	1240 (17.44)	32 (8.21)	
Mean ± SD	8.42 ± 5.19	8.66 ± 5.01	6.65 ± 4.83	< 0.001	6.42 ± 5.13	7.63 ± 5.00	5.79 ± 4.57	< 0.001	8.68 ± 5.15	8.79 ± 5.00	6.83 ± 4.87	< 0.001
Physical activity. MET												
Mean ± SD	39.85 ± 5.76	38.81 ± 6.37	35.24 ± 4.36	< 0.001	38.54 ± 4.98	37.07 ± 4.77	34.35 ± 4.44	< 0.001	40.01 ± 5.83	39.02 ± 6.50	35.42 ± 4.33	< 0.001
BMI- no. (%)												
< 25	394 (25.42)	2354 (29.60)	134 (28.51)	0.001	36 (20.34)	216 (25.50)	20 (25.00)	0.497	358 (26.07)	2138 (30.08)	114 (29.23)	0.002
25–29.9	633 (40.84)	3272 (41.14)	183 (38.94)		80 (45.20)	337 (39.79)	29 (36.25)		553 (40.28)	2935 (41.30)	154 (39.49)	
≥ 30	523 (33.74)	2328 (29.27)	153 (32.55)		61 (34.46)	294 (34.71)	31 (38.75)		462 (33.65)	2034 (28.62)	122 (31.28)	
Mean ± SD	28.28 ± 5.07	27.71 ± 4.88	28.13 ± 5.06	< 0.001	28.78 ± 4.62	28.40 ± 5.04	28.92 ± 5.05	0.48	28.21 ± 5.13	27.62 ± 4.85	27.97 ± 5.05	< 0.001
Abdominal obesity- no. (%)												
Yes	857 (55.33)	4214 (52.99)	306 (65.11)	< 0.001	123 (69.49)	545 (64.34)	56 (70.00)	0.292	734 (53.50)	3669 (51.64)	250 (64.10)	< 0.001
No	692 (44.67)	3738 (47.01)	164 (34.89)		54 (30.51)	302 (35.66)	24 (30.00)		638 (46.50)	3436 (48.36)	140 (35.90)	
Mean ± SD	96.98 ± 11.57	95.65 ± 11.47	96.79 ± 11.57	< 0.001	98.55 ± 10.33	97.62 ± 11.54	98.60 ± 11.55	< 0.001	96.77 ± 11.71	95.41 ± 11.44	96.43 ± 11.55	< 0.001
Smoking- no. (%)												
Yes	404 (26.27)	2026 (25.63)	113 (24.20)	0.662	43 (24.29)	232 (27.42)	22 (27.50)	0.690	361 (26.52)	1794 (25.42)	91 (23.51)	0.454
No	1134 (73.73)	5878 (74.37)	354 (75.80)		134 (75.71)	614 (72.58)	58 (72.50)		1000 (73.48)	5264 (74.58)	296 (76.49)	
Alcohol consumption- no. (%)												
Yes	165 (10.73)	803 (10.16)	25 (5.35)	0.002	7 (3.95)	73 (8.63)	3 (3.75)	0.042	158 (11.61)	730 (10.34)	22 (5.68)	0.003
No	1373 (89.27)	7101 (89.84)	442 (94.65)		170 (96.05)	773 (91.37)	77 (96.25)		1203 (88.39)	6328 (89.66)	365 (94.32)	
Opium consumption- no. (%)												
Yes	373 (24.25)	1839 (23.27)	133 (28.48)	0.030	45 (25.42)	212 (25.06)	26 (32.50)	0.345	328 (24.10)	1627 (23.05)	107 (27.65)	0.093
No	1165 (75.75)	6065 (76.73)	334 (71.52)		32 (74.58)	634 (74.94)	54 (67.50)		1033 (75.90)	5431 (76.95)	280 (72.35)	

*WSI* wealth score index, *BMI* body mass index, *MET* metabolic equivalent of task.

Table 2 presents the frequency of non-communicable diseases by sleep duration in HDU and NHDU groups. In the total population and NHDU group, the prevalence of diabetes (P <  < 0.001), Thyroid disease (P = 0.045), MI (P = 0.001), CHD (P = 0.002), depression, and hypertension (P < 0.001) were higher in people with LSD (> 9 h). Also, in the HDU group, the prevalence of Thyroid disease (P = 0.011) was higher in LSD (> 9 h) (Table 2).

**Table 2:** Distribution of diseases of the participant taking hypnotic drug or non, by sleep duration in RCS.

Variable	Sleep duration											
	Total (n = 9981)				Hypnotic drug user (n = 1105)				Non-hypnotic drug user (8876)			
	< 6 h (n = 1552)	6–9 h (n = 7959)	> 9 h (n = 470)	P-value	< 6 h (n = 177)	6–9 h (n = 848)	> 9 h (n = 80)	P-value	< 6 h (n = 1375)	6–9 h (n = 7111)	> 9 h (n = 390)	P-value
Diabetes- no. (%)												
Yes	328 (21.26)	1472 (18.59)	132 (28.21)	< 0.001	50 (28.25)	238 (28.07)	26 (32.50)	0.701	278 (20.35)	1234 (17.46)	106 (27.32)	< 0.001
No	1215 (78.74)	6445 (81.41)	336 (71.79)		127 (71.75)	610 (71.93)	54 (67.50)		1088 (79.65)	5835 (82.54)	282 (72.68)	
Dyslipidemia**-**no. (%)												
Yes	1155 (75.00)	5766 (72.82)	357 (76.62)	0.055	140 (79.55)	676 (80.00)	70 (87.50)	0.255	1015 (74.41)	5090 (71.96)	287 (74.35)	0.124
No	385 (25.00)	2152 (27.18)	109 (23.39)		36 (20.45)	169 (20.00)	10 (12.50)		349 (25.59)	1983 (28.04)	99 (25.65)	
Fatty liver- no. (%)												
Yes	183 (11.86)	781 (9.86)	48 (10.26)	0.060	29 (16.38)	120 (14.15)	14 (17.50)	0.577	154 (11.27)	661 (9.35)	34 (8.76)	0.074
No	1360 (88.14)	7136 (90.14)	420 (89.74)		148 (83.62)	728 (85.85)	66 (82.50)		1212 (88.73)	6408 (90.65)	354 (91.24)	
Thyroid- no. (%)												
Yes	131 (8.49)	710 (8.97)	57 (12.18)	0.045	16 (9.04)	107 (12.62)	18 (22.50)	0.011	115 (8.42)	603 (8.53)	39 (10.05)	0.566
No	1412 (91.51)	7207 (91.03)	411 (87.82)		161 (90.96)	741 (87.38)	62 (77.50)		1251 (91.58)	6466 (91.47)	349 (89.95)	
Chronic lung disease- no. (%)												
Yes	52 (3.37)	193 (2.44)	14 (2.99)	0.095	9 (5.08)	25 (2.95)	0 (0)	0.083	43 (3.15)	168 (2.38)	14 (3.61)	0.102
No	1491 (96.63)	7724 (97.56)	454 (97.01)		167 (94.99)	823 (97.05)	80 (100)		1323 (96.85)	6901 (97.62)	374 (96.39)	
MI- no. (%)												
Yes	52 (3.37)	217 (2.74)	27 (5.77)	0.001	9 (5.08)	42 (4.95)	7 (8.75)	0.345	43 (3.15)	175 (2.48)	20 (5.15)	0.004
No	1491 (96.63)	7700 (97.26)	441 (94.23)		168 (94.92)	806 (95.05)	73 (91.25)		1323 (96.85)	6894 (97.52)	368 (94.85)	
CHD- no. (%)												
Yes	157 (10.17)	656 (8.29)	57 (12.18)	0.002	37 (20.90)	149 (17.57)	16 (20)	0.533	120 (8.78)	507 (7.17)	41 (10.57)	0.009
No	1386 (89.83)	7261 (91.71)	411 (87.82)		140 (79.10)	699 (82.43)	64 (80)		1246 (91.22)	6562 (92.83)	347 (89.43)	
Stroke- no. (%)												
Yes	20 (1.30)	120 (1.52)	12 (2.56)	0.143	7 (3.95)	24 (2.83)	5 (6.25)	0.219	13 (0.95)	96 (1.36)	7 (1.80)	0.332
No	1523 (98.70)	7797 (98.48)	456 (97.44)		170 (96.05)	824 (97.17)	75 (93.75)		1353 (99.05)	6973 (98.64)	381 (98.20)	
Multiple Sclerosis- no. (%)												
Yes	2 (0.13)	15 (0.19)	0 (0)	0.573	1 (0.56)	4 (047)	0 (0)	0.810	1 (0.07)	11 (0.16)	0 (0)	0.570
No	1541 (99.87)	7902 (99.81)	468 (100)		176 (99.44)	844 (99.53)	80 (100)		1365 (99.93)	7058 (99.84)	388 (100)	
Depression- no. (%)												
Yes	379 (24.56)	1743 (22.02)	141 (30.13)	< 0.001	124 (70.06)	532 (62.74)	55 (68.75)	0.126	255 (18.67)	1211 (17.13)	86 (22.16)	0.021
No	1164 (75.44)	6174 (77.98)	327 (69.87)		53 (29.94)	316 (37.26)	25 (31.25)		1111 (81.33)	5858 (82.87)	302 (77.84)	
Epilepsy- no. (%)												
Yes	24 (1.56)	102 (1.29)	8 (1.71)	0.557	6 (3.39)	26 (3.07)	0 (0)	0.269	18 (1.32)	76 (1.08)	8 (2.06)	0.173
No	1519 (98.44)	7815 (98.71)	460 (98.29)		171 (96.61)	822 (96.93)	80 (100)		1348 (98.68)	6993 (98.92)	380 (97.94)	
Recurrent headache- no. (%)												
Yes	470 (30.46)	2284 (28.85)	135 (28.85)	0.439	68 (38.42)	335 (39.50)	32 (40.00)	0.957	402 (29.43)	1949 (27.57)	103 (26.55)	0317
No	1073 (69.54)	5634 (71.15)	333 (71.15)		109 (61.58)	513 (60.50)	48 (60.00)		964 (70.57)	5121 (72.43)	285 (73.45)	
Chronic headache- no. (%)												
Yes	124 (8.04)	573 (7.24)	44 (9.40)	0.145	30 (16.95)	133 (15.68)	13 (16.25)	0.913	94 (6.88)	440 (6.22)	31 (7.99)	0.283
No	1419 (91.96)	7344 (92.76)	424 (90.60)		147 (83.05)	715 (84.32)	67 (83.75)		1272 (93.12)	6629 (93.78)	357 (92.01)	
Hypertension- no. (%)												
Yes	390 (25.28)	1710 (21.60)	135 (28.85)	< 0.001	75 (42.37)	339 (39.98)	33 (41.25)	0.830	315 (23.06)	1371 (19.39)	102 (26.29)	< 0.001
No	1153 (74.72)	6207 (78.40)	333 (71.15)		102 (57.63)	509 (60.02)	47 (58.75)		1551 (76.94)	5698 (80.61)	286 (73.71)	

*MI* myocardial ischemia, *CHD* chronic heart diseases.

Furthermore, the odds ratios of selected variables related to HDU and NHDU groups in different sleep duration groups are reported in Table 3. According to the results of backward logistic regression analysis, the odds of SSD (< 6 h) increased by increasing the age (OR:1.02 (95% CI 1.015–1.03), P < 0.001), BMI (OR:1.03 (95% CI 1.02–1.04), P < 0.001), physical activity (OR: 1.03 (95% CI 1.02–1.04), P < 0.001) and depression (OR: 1.17 (95% CI 1.02–1.33), P = 0.023) in total population compared to the reference group. We observed that in the total population, people with MI had increased odds of LSD (OR: 1.66 (95% CI 1.01–2.72), P = 0.045). Alcohol consumption also showed a protective effect on LSD in all individuals (OR: 0.59 (95% CI 0.37–0.94), P = 0.027) (Table 3).

**Table 3:** Odds ratios (with 95% CIs) of long and short sleep duration in hypnotic drug users and non-users in relation to demographic factors, habits, and medical history.

Variable	Multivariate						
	Sleep duration	Total	P-value	Hypnotic drug user	P-value	Non-Hypnotic drug user	P-value
Age	6–9	1		1		1	
	< 6	1.02 (1.02–1.03)	< 0.001	1.01 (0.99–1.04)	0.287	1.02 (1.02–1.03)	< 0.001
	> 9	1.01 (0.99–1.02)	0.151	0.99 (0.96–1.03)	0.778	1.01 (0.99–1.02)	0.163
Female	6–9	1		1		1	
	< 6	0.99 (0.85–1.16)	0.899	1.07 (0.60–1.94)	0.812	0.99 (0.84–1.16)	0.884
	> 9	3.03 (2.18–4.20)	< 0.001	1.31 (0.56–3.11)	0.534	3.43 (2.39–4.91)	< 0.001
WSI	6–9	1		1		1	
	< 6	0.99 (0.93–1.06)	0.862	1.07 (0.88–1.31)	0.482	0.98 (0.92–1.06)	0.649
	> 9	0.91 (0.82–1.02)	0.117	0.99 (0.75–1.31)	0.941	0.90 (0.80–1.02)	0.103
Education	6–9	1		1		1	
	< 6	1.01 (1.00–1.03)	0.058	0.96 (0.92–1.00)	0.077	1.02 (1.01–1.04)	0.006
	> 9	0.96 (0.94–0.99)	0.007	0.93 (0.87–0.99)	0.034	0.97 (0.94–0.99)	0.037
BMI	6–9	1		1		1	
	< 6	1.03 (1.02–1.04)	< 0.001	1.02 (0.98–1.06)	0.362	1.03 (1.02–1.05)	< 0.001
	> 9	0.98 (0.96–1.00)	0.091	0.99 (0.93–1.04)	0.608	0.98 (0.96–1.00)	0.014
Physical activity	6–9	1		1		1	
	< 6	1.03 (1.02–1.04)	< 0.001	1.06 (1.03–1.10)	< 0.001	1.03 (1.02–1.04)	< 0.001
	> 9	0.83 (0.81–0.85)	< 0.001	0.82 (0.76–0.88)	< 0.001	0.83 (0.81–0.86)	< 0.001
Opium consumption	6–9	1		1		1	
	< 6	1.04 (0.87–1.23)	0.663	1.41 (0.83–2.41)	0.208	1.01 (0.84–1.21)	0.910
	> 9	2.15 (1.59–2.92)	< 0.001	2.13 (1.01–4.46)	0.046	2.16 (1.54–3.02)	< 0.001
Alcohol consumption	6–9	1		1		1	
	< 6	1.19 (0.97–1.45)	0.100	0.51 (0.21–1.20)	0.124	1.28 (1.04–1.58)	0.022
	> 9	0.59 (0.37–0.94)	0.027	0.34 (0.09–1.22)	0.099	0.66 (0.40–1.09)	0.106
Smoking	6–9	1		1		1	
	< 6	0.95 (0.80–1.14)	0.603	0.85 (0.46–1.56)	0.593	0.97 (0.81–1.17)	0.758
	> 9	1.13 (0.80–1.59)	0.478	0.73 (0.31–1.71)	0.466	1.20 (0.82–1.75)	0.355
Diabetes	6–9	1		1		1	
	< 6	1.03 (0.89–1.20)	0.669	0.91 (0.61–1.35)	0.647	1.06 (0.90–1.24)	0.491
	> 9	1.13 (0.89–1.44)	0.300	1.07 (0.61–1.86)	0.813	1.16 (0.89–1.51)	0.278
Thyroid	6–9	1		1		1	
	< 6	0.91 (0.75–1.12)	0.376	0.65 (0.37–1.15)	0.139	0.96 (0.78–1.20)	0.735
	> 9	1.19 (0.88–1.61)	0.265	1.96 (1.04–3.67)	0.037	1.01 (0.70–1.44)	0.970
MI	6–9	1		1		1	
	< 6	1.11 (0.78–1.57)	0.574	1.08 (0.47–2.46)	0.860	1.13 (077–1.69)	0.534
	> 9	1.66 (1.01–2.72)	0.045	1.94 (0.71–5.34)	0.199	1.56 (0.88–2.76)	0.131
CHD	6–9	1		1		1	
	< 6	1.08 (0.87–1.34)	0.481	1.16 (0.73–1.85)	0.518	1.05 (0.82–1.34)	0.699
	> 9	0.85 (0.60–1.21)	0.361	0.70 (0.34–1.41)	0.311	0.91 (0.61–1.37)	0.659
Stroke	6–9	1		1		1	
	< 6	0.68 (0.41–1.11)	0.124	1.25 (0.51–3.08)	0.626	0.55 (0.30–1.01)	0.054
	> 9	1.04 (0.55–1.98)	0.903	1.72 (0.56–5.30)	0.342	0.81 (0.35–1.83)	0.608
Depression	6–9	1		1		1	
	< 6	1.17 (1.02–1.33)	0.023	1.45 (1.01–2.09)	0.049	1.14 (0.97–1.33)	0.104
	> 9	1.24 (0.99–1.54)	0.056	1.21 (0.71–2.07)	0.480	1.22 (0.94–1.58)	0.136
Dyslipidemia	6–9	1		1		1	
	< 6	1.01 (0.89–1.15)	0.874	0.99 (0.64–1.52)	0.952	1.02 (0.88–1.17)	0.829
	> 9	0.95 (0.75–1.20)	0.657	1.41 (0.68–2.92)	0.358	0.89 (0.69–1.15)	0.377
Fatty liver	6–9	1		1		1	
	< 6	1.16 (0.98–1.39)	0.092	1.16 (0.72–1.85)	0.541	1.15 (0.95–1.39)	0.153
	> 9	1.00 (0.73–1.39)	0.985	1.45 (0.74–2.82)	0.276	0.89 (0.61–1.30)	0.540
Lung disease	6–9	1		1		1	
	< 6	1.33 (0.97–1.83)	0.076	1.77 (0.79–3.97)	0.167	1.27 (0.90–1.79)	0.179
	> 9	0.88 (0.50–1.58)	0.675	NA*	0.986	1.15 (0.64–2.07)	0.641
Hypertension	6–9	1		1		1	
	< 6	1.01 (0.87–1.17)	0.923	0.94 (0.63–1.39)	0.751	1.02 (0.87–1.20)	0.787
	> 9	0.91 (0.71–1.17)	0.482	0.81 (0.45–1.43)	0.461	0.96 (0.73–1.27)	0.800

*Cannot be calculated because the number of people in this category is zero.

*WSI*: wealth score index, *BMI* body mass index, *MI* myocardial ischemia, *CHD* chronic heart diseases.

In the NHDU group, age (OR:1.02 (95% CI 1.02–1.03), P < 0.001), education (OR: 1.02 (95% CI 1.01–1.04), P = 0.006), BMI (OR: 1.03 (95% CI 1.02–1.05), P < 0.001), physical activity (OR: 1.03 (95% CI 1.02–1.04), P < 0.001), and alcohol consumption (OR: 1.28 (95% CI 1.04–1.58), P = 0.022) were related to increased odds of SSD (Table 3). In the total population and HDU group, depression showed a significantly increased OR for SSD (total OR: 1.17 (95% CI 1.02–1.33), P = 0.023, HDU group OR: 1.45 (95% CI 1.01–2.09), P = 0.049). In the total population, the odds of LSD were significantly higher in women (OR: 3.03 (95% CI 2.18–4.2), P < 0.001) compared to men, and this higher ratio was also observed in the NHDU group (OR: 3.43 (95% CI 2.39–4.91), P < 0.001). In the HDU group, the odds of over LSD (> 9 h) increased in people with thyroid disease (OR: 1.96 (95% CI 1.04–3.67), P = 0.037) (Table 3).

In total subjects, HDU and NHDU groups, opium use was related to increased odds of LSD (OR: 2.15 (95% CI 1.59–2.92), P < 0.001, OR: 2.13 (95% CI 1.01–4.46), P = 0.046, and OR: 2.16 (95% CI 1.54–3.02), P < 0.001, respectively). Higher education also showed a protective effect on LSD in all individuals (OR: 0.96 (95% CI 0.94–0.99), P = 0.007), HDU group (OR: 0.93 (95% CI 0.87–0.99), P = 0.034) and NHDU groups (OR: 0.97 (95% CI 0.94–0.99), P = 0.037). Moreover, in all three groups with increasing physical activity, the odds of SSD (< 6 h) increased (P < 0.001), and the odds of LSD (> 9 h) decreased (P < 0.001) (Table 3).

## Discussion

The findings of the present study showed that a higher odds ratio of SSD (< 6 h) is significantly associated with age, higher education level, BMI, physical activity, alcohol consumption, and depression. LSD (> 9 h), on the other hand, showed a positive association with age, female sex, opium consumption, and history of MI, and a reverse association with higher education, physical activity, and alcohol consumption.

For more accurate results, we performed a sensitivity analysis, stratifying for hypnotic drug use in our study population to assess whether hypnotic drug use may impact the association of sleep duration with risk factors and the related medical conditions. In this study, women slept more than men. These findings support objective and subjective reports documenting longer sleep duration among women^28,29^. Our sensitivity analysis showed that in the HDU group, LSD is not associated with the female sex suggesting the gender differential impact of hypnotic drugs. It has been demonstrated by previous studies that the frequency of sleep duration is sex-dependent, and they are more prevalent among females. The average total sleep time (TST) was significantly longer in women^30^. The results of the present study confirm that LSD (> 9 h) is three times more likely in women compared to men, and additionally indicate a sex differential impact of HDU in adults.

The close relationship between sleep duration and depression and anxiety is previously demonstrated^31–35^. Interestingly, here we found a significant 45% increased odds ratio of SSD (< 6 h) associated with depression and a 96% increased odds ratio of LSD (> 9 h) in Thyroid diseases, only in the HDU group, and these associations were not significant in the NHDU group. Considering this is a cross-sectional study, it is suggested that in the follow-up phase of the study, this association is further investigated.

We observed 28% higher odds of SSD (< 6 h) associated with alcohol consumption in the NHDU group. This finding is in line with recent reports that indicated worse sleeping patterns with alcohol drinking habits, such as having trouble staying asleep and frequent wakening during the night, shorter duration of sleep, and snoring^36–38^. Stratifying for the HDU group, we found a reverse relationship and a 41% decreased odds ratio of LSD in alcohol consumers. We suggest future studies that assess whether hypnotic drugs may alleviate the adverse sleep duration consequences of drinking habits in adults.

Controversial reports have been published regarding the association or lack of association between physical activity and odds of sleep disturbances, which may be explained by the type and duration of the physical activity measured in different studies, and the impact of variation in demographic and socioeconomic factors in other study populations^39–46^. Findings from our study showed physical activity to be slightly associated with LSD (< 6 h) (around 3% increased odds ratio) and inversely connected to approximately 20% lower odds ratio of LSD (> 9 h). This relationship was observed in the HDU group.

Our results showed that chronic opium use is associated with a more than doubled odds ratio of LSD similarly in HDU and NHDU groups. Abnormal sleep duration due to chronic substance use has been previously demonstrated^47–51^. In the present study, we further assessed whether hypnotic drug use might alleviate or exacerbate this association. Our statistical analyses do not support the significant impact of hypnotic drug use on the association of sleep duration and opium consumption.

A significant association is reported between SSD and BMI in some previous cross-sectional and longitudinal studies^52,53^. However, due to the contrasting results of previous studies that indicate a beneficial or harmful impact of LSD on obesity, the link between LSD and BMI is not well defined^52^. Our results confirm the association between SSD and higher BMI but did not support a positive relationship between LSD and obesity.

Previous studies have demonstrated contradictory results on the relationship between sleep duration and education levels. Some found LSD associated with higher education, and some did not find a significant association between them^54–56^. Our results showed a negative association between education levels and sleep duration in the RCS population, as more education was positively associated with SSD and negatively associated with LSD.

In the present study, it was shown that sleep duration decreases with age. Therefore, we may further emphasize the importance of considering the age group when assessing the relation of sleep duration with diseases and all-cause mortality. Since advanced age is itself associated with higher mortality, and this may affect the connection of sleep duration with mortality^57–59^. For example, a Swedish prospective cohort study on 43,863 individuals (64% women) found that both SSD and LSD are associated with mortality only among young individuals, and in participants older than 65, they did not find an association between abnormal sleep duration and mortality when considering the impact of age in their analysis^60^.

Previous observational studies have indicated both SSD and LSD are positively associated with MI and CVD, suggesting abnormal sleep duration is a potent risk factor for CVD^61–63^. Here, we found LSD to be associated with MI. However, our findings do not support a statistically significant relationship between SSD and MI. On the other hand, our study did not find a significant association between abnormal sleep duration and CHD.

Some previous reports demonstrated an association between both SSD and LSD with an increase in the risk of thyroid dysfunction, suggesting that abnormal sleep duration may exert a detrimental impact on thyroid function leading to an increased risk of subclinical thyroid problems^64,65^. The present study found significantly increased odds of thyroid dysfunction associated with LSD only among the NHDU group. Regarding SSD, our data does not indicate a significant relationship between sleep duration and thyroid diseases.

The strength points of the present study are the stratification for hypnotic drug use when analyzing the association of sleep duration with the related risk factors and diseases. Additionally, a comprehensive cohort study performed allowed us to assess the relationship between sleep duration with multiple illnesses (depression, MI, CHD, diabetes, etc.) and lifestyle factors such as opium use, alcohol consumption, and physical activity in multivariate regression models, based on the data obtained in the RCS cohort. Additionally, computer-assisted, server-based face-to-face interviews in the RCS cohort studies performed by trained healthcare experts have increased the quality and accuracy of data collection. However, a limitation of this study is that data on smoking, opium use, sleep duration, and medical history of diseases is based on self-reports of participants and are not based on clinical assessment and sleep laboratory data, which may have entered some levels of misclassification due to self-reporting and recall biases. Another limitation was the cross-sectional design of the study. In contrast, did not allow for deriving any causal inferences. Accordingly, this relationship will be reconsidered in the follow-up phase of this prospective study.

## Conclusion

Overall, our results indicated demographic and lifestyle factors such as age, BMI, education, physical activity, alcohol consumption, and depression to be associated with SSD. Age, female sex, opium consumption, and history of MI displayed a significant association with higher odds of LSD, while education, physical activity, and alcohol consumption were associated with lower odds of LSD. Our sensitivity analyses showed a connection between thyroid dysfunction and depression with SSD only in hypnotic drug users, suggesting that more caution needs to be taken before hypnotic drug administration.

## Data Availability

The current study’s data are available at the PERSIAN Adult Cohort Study Center, Rafsanjan University of Medical Sciences, Iran. The data are not available publicly. However, upon reasonable request, the data can be obtained from the corresponding author (Fatemeh Ayoobi, ayoobi.fatemeh@gmail.com).

## Abbreviations

LSD: Long sleep duration
SSD: Short sleep duration
NSD: Normal sleep duration
RCS: Rafsanjan cohort study
PERSIAN: Prospective epidemiological research studies in Iran
BMI: Body mass index
MI: Myocardial ischemia
CHD: Chronic heart diseases
RLS: Restless leg syndrome
CVD: Cardiovascular diseases
HTN: Hypertension
BDZ: Benzodiazepines
MET: Metabolic equivalent of task
ORs: Odds ratios
WSI: Wealth score index
HDU: Hypnotic drug users
NHDU: Non-hypnotic drug users
